# Poly(ethylene-imine)-Functionalized Magnetite Nanoparticles Derivatized with Folic Acid: Heating and Targeting Properties

**DOI:** 10.3390/polym13101599

**Published:** 2021-05-15

**Authors:** Mariano Ortega-Muñoz, Simona Plesselova, Angel V. Delgado, Francisco Santoyo-Gonzalez, Rafael Salto-Gonzalez, Maria Dolores Giron-Gonzalez, Guillermo R. Iglesias, Francisco Javier López-Jaramillo

**Affiliations:** 1 Department of Organic Chemistry, Institute of Biotechnology, Faculty of Sciences, University of Granada, 18071 Granada, Spain; mortegam@ugr.es (M.O.-M.); fsantoyo@ugr.es (F.S.-G.); 2 Department of Biochemistry and Molecular Biology II, Faculty of Pharmacy, University of Granada, 18071 Granada, Spain; s.plesselova@gmail.com (S.P.); rsalto@ugr.es (R.S.-G.); mgiron@ugr.es (M.D.G.-G.); 3 Department of Applied Physics, Faculty of Sciences, University of Granada, 18071 Granada, Spain; adelgado@ugr.es

**Keywords:** alternating magnetic field, cell viability, folic acid, magnetic hyperthermia, magnetite, poly(ethylene-imine), nanotoxicity

## Abstract

Magnetite nanoparticles (MNPs) coated by branched poly (ethylene-imine) (PEI) were synthesized in a one-pot. Three molecular weights of PEI were tested, namely, 1.8 kDa (sample **MNP-1**), 10 kDa (sample **MNP-2**), and 25 kDa (sample **MNP-3**). The **MNP-1** particles were further functionalized with folic acid (FA) (sample **MNP-4**). The four types of particles were found to behave magnetically as superparamagnetic, with **MNP-1** showing the highest magnetization saturation. The particles were evaluated as possible hyperthermia agents by subjecting them to magnetic fields of 12 kA/m strength and frequencies ranging between 115 and 175 kHz. **MNP-1** released the maximum heating power, reaching 330 W/g at the highest frequency, in the high side of reported values for spherical MNPs. In vitro cell viability assays of **MNP-1** and **MNP-4** against three cell lines expressing different levels of FA receptors (FR), namely, HEK (low expression), and HeLa (high expression), and HepG2 (high expression), demonstrated that they are not cytotoxic. When the cells were incubated in the presence of a 175 kHz magnetic field, a significant reduction in cell viability and clone formation was obtained for the high expressing FR cells incubated with **MNP-4**, suggesting that **MNP-4** particles are good candidates for magnetic field hyperthermia and active targeting.

## 1. Introduction

Cancer remains one of the most challenging diseases, its treatment involving, when possible, a combination of surgical resection of the tumor with radiation therapy, chemotherapy, and/or immunotherapy. However, this approach does not guarantee the complete eradication of the cancer, and it is not exempt from risk since normal tissues are also damaged. Alternative therapies are in demand, and the long-known positive effects of thermal therapy on cancer has gained attention. The efficiency of the thermal treatment is dependent on both the magnitude and the extent of the temperature increment [1,2]. The range of 41–48 °C is the so-called relevant temperature range suitable for hyperthermia treatments.

The underlying biological processes induced by hyperthermia are not fully understood and the differential thermal sensitivity of tumor cells remains controversial [3,4,5,6], despite the efforts dedicated to study the mechanism of the cellular effects [7]. Protein denaturation starts at 39 °C, and at 40 °C, cells enter into an inactivation process that lasts for several hours; the surviving cells reach a temporary thermotolerance that is overcome when the temperature rises to 41–42 °C for several hours [8]. Severe hyperthermia treatments (43–45 °C) provoke oxidative stress, and irreversible injury occurs when the temperature reaches values above 48 °C for a period of time exceeding a few minutes [1]. 

While tumor ablation is feasible, hyperthermia is generally implemented in the context of cancer treatment at moderate temperatures (41–42 °C) as part of a combined therapy, and its effect is the modification of the response to some drugs and/or sensitivity to X-ray irradiation, increasing the efficiency of the traditional treatments and reducing their side effects [9,10]. Regardless of the final temperature reached, hyperthermia implies the heating of specific areas and the production of well-defined hot spots. In this context, nanotechnology offers alternatives that are based on nanoparticles smaller than 100 nm that are biocompatible and able to produce heat [2]. 

The proposed approach, known as magnetic fluid hyperthermia (MFHT), involves the selective administration of MNPs and the exposition of the patient to an alternating magnetic field (AMF), that provokes rotation of the particles (Brownian rotation) and rotation of the magnetic moment in the particle (Neel’s rotation) with the concomitant heat generation [11,12]. Currently, several clinical trials are in progress for prostate cancer and glioblastoma [13], and some have been reported, using licensed iron oxide nanoparticles (IONPs) and the medical AMF device NanoActivator^®^ (MagForce AG, Berlin, Germany) [13,14].

The limitations imposed by eddy currents and the fact that the implementation of MNPs in therapy is affected by features such as shape, size, morphology, or dispersibility have boosted the study of magnetic particles with this purpose [15]. In particular, superparamagnetic iron oxide nanoparticles (SPIONs) have become the preferred materials due to their relative ease of synthesis by coprecipitation, hydrothermal, pyrolysis, sol–gel, microemulsion, sonochemical, electrodeposition, or polyol methods, as well as physical ones [16]. However, they are prone to agglomeration and oxidation in the physiological environment, their fate in the human body being highly dependent on their surface properties. Hence, surface modification is applied to improve their physicochemical and mechanical properties and to control their surface activity, biocompatibility, and dispersibility. The surface can be functionalized with organic or inorganic molecules, and the functionalization can be implemented during the synthesis or post -synthesis of the SPION. The chemistry can be covalent, noncovalent, or be based on just sorption [16,17,18,19]. Among the different modifications, those aimed at promoting the delivery of the particles to the site of action are of paramount importance for localizing and optimizing the temperature increase. The fact that the folic acid receptor (FR) is overexpressed in many cancers has led to the investigation of the feasibility of using IONPs functionalized with folic acid (FA) for receptor-mediated (or active) targeting [20,21,22,23,24,25,26,27,28].

Polyethyleneimine (PEI) is a commercial polymer widely used for different purposes, from biocompatible coating of a surface to gene delivery [29,30,31]. It consists of repeating units of amino groups spaced by two aliphatic carbons to yield a linear polymer of secondary amines or a branched polymer that contains primary, secondary, and tertiary amino group. As a procedure for functionalizing MNPs, the solvothermal synthesis of Fe_3_O_4_ has been reported using PEI to prevent aggregation and decorate the MNP surface with amino groups that are intended, for instance, (i) to reduce HAuCl_4_ and yield Fe_3_O_4_/Au core shell [32,33,34,35,36], (ii) to prepare Yb^3+^ magnetic nanocomposites [37], (iii) as a coinitiator in photopolymerization [37]. The use of amino groups with that purpose has been widely described; for instance, Hanafy et al. [38] coated magnetite with PEG-bis amine for subsequent adsorption of carboxymethyl cellulose. The procedure proposed in the present work has the advantage that the amino groups of PEI are incorporated during the synthesis and not post-synthesis, likely yielding a more stable coating. This has also been done by other authors, such as Felix et al. [36]. However, to the best of our knowledge, these particles have not been studied from the perspective of their biomedical application and the surface amino groups have not been exploited to incorporate targeting ligands. Such a study is described in the present paper. Herein we report the synthesis and characterization of Fe_3_O_4_-PEI nanoparticles, their covalent functionalization with FA, and the analysis of their feasibility for MFHT. The results show that these particles provide excellent performance in MFHT in moderate magnetic fields and show low cytotoxicity, features which make them suitable as a coadjutant in cancer treatment through hyperthermia.

## 2. Materials and Methods

### 2.1. Materials

The chemicals used for the synthesis were provided by Sigma-Aldrich (Darmstadt, Germany) (PEI2K, PEI10K, PEI25K, DCC, Cl_3_Fe·6H_2_O, ethane-1,2-diol, folic acid), and Scharlau (Barcelona, Spain) (anhydrous DMF, anhydrous pyridine, sodium acetate), and they were used as received. Water used in the experiments was deionized and filtered in a Milli-Q Academic, Millipore (Molsheim, France) system.

### 2.2. Methods

**Scheme 1:**
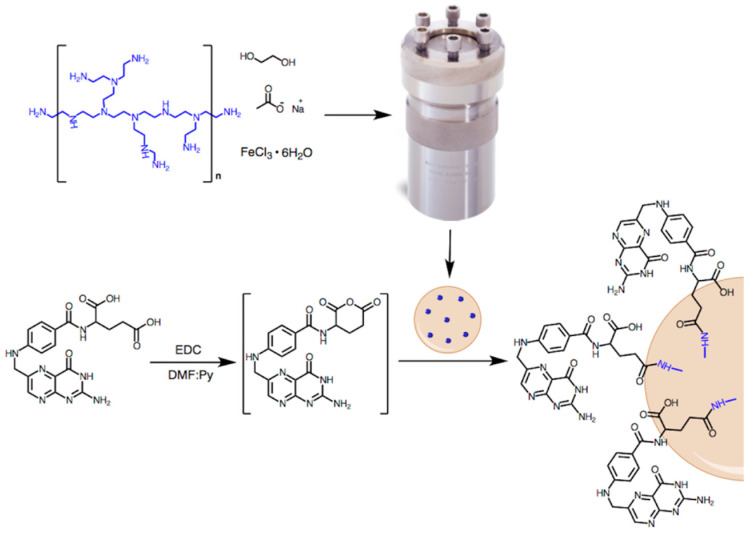
The process of linking folic acid (FA, bottom left) to the magnetite nanoparticles (bottom, right). FA was reacted with N,N′-dicyclohexylcarbodiimide (EDC)/dimethyl formamide-pyridine (DMF-Py), and the resulting anhydride was contacted with PEI-coated magnetite (top).

The synthesis of the particles was performed as described by Wang et al. [35]. Branched PEI with molecular weights of 1.8 kDa (PEI2K), 10 kDa (PEI10K), and 25 kDa (PEI25K), and containing primary, secondary, and tertiary amino groups were used, yielding samples **MNP-1**, **MNP-2**, and **MNP-3**, respectively. An amount of 1 g of FeCl_3_·6H_2_O, 2 g of branched PEI and 2 g of sodium acetate, and 60 mL of ethane-1,2-diol was sonicated for 30 min and then stored overnight at 40 °C. After a new sonication for 30 min, the sample was heated at 60 °C under stirring for 1 h. Next, the sample was sealed in a 125 mL acid digestion vessel and incubated in an oven at 200 °C for 10 h. Then, nanoparticles were separated with the help of a permanent magnet, redispersed in deionized water, and dried at 60 °C under vacuum (300 mbar). The functionalization with FA (sample **MNP-4**) was carried out in one pot (see Scheme 1) and protected from light. An amount of 0.2 mmol of FA was reacted in 1.2 mmol of N,N′-dicyclohexylcarbodiimide (DCC) in 7.5 mL of DMF: pyridine (5:1) for 30 min to yield folic acid anhydride [39]. Then, 90 mg of nanoparticles were added to the reaction that, after sonication, was were allowed to proceed at room temperature for 19 h. Nanoparticles were washed thoroughly, first with methanol, and then with ether and dried in air, protected from light.

Particle size, shape, and EDX analyses were performed using a FEI-Titan (ThermoScientific, Hillsboro, OR, USA) high-resolution transmission electron microscope (HRTEM) operated at 300 kV. Furthermore, hydrodynamic particle diameters were evaluated by dynamic light scattering in a Malvern Zeta Sizer NanoZS (Malvern Instruments, Malvern, UK). FTIR spectra were recorded at room temperature from powder using a PerkinElemer FT-IR Spectrum Two. (Waltham, MA, USA) X-ray photoelectron spectroscopy (XPS) analysis of the samples was performed in a Kratos Axis Ultra-DLD (Kratos Analytical, Manchester, UK) XPS spectrometer using monochromatic Al Kα radiation and data were collected from three spectra from a 300 μm × 700 μm area.

All particles described in this work demonstrated to be colloidally stable, with no sedimentation for days after preparation. As an indication of the stability, we measured the electrophoretic mobility using the above cited Malvern instrument. No attempt was made to calculate the zeta potential because it lacks true significance when the particles are coated with polymers, as in our case [40]. A sufficiently high (roughly around 3 μm·s^−1^/V·cm^−1^) is a good indication of electrostatic repulsion between the particles contributing to their stability.

Magnetic hyperthermia was applied to samples contained in 1.5 mL Eppendorf tubes, which were located inside a coil (8 turns, 45 mm coil length, 20 mm inner diameter) built with a copper tube of 6 mm inner diameter. Thermostatic water was circulated through the tube during the experiment, in order to avoid interference of Joule heating on the hyperthermia induced by the AMF. A Royer-type oscillator was used to produce the alternating current applied to the coil for producing the AMF. The frequencies used were 115, 135, 155, and 175 kHz, and the *H* field amplitude was 12 kA/m in all cases, as determined with a NanoScience Laboratories Ltd. Probe (Keele, UK). The tube containing the suspension (0.5 mL and 10 mg/mL particle concentration) was pre-thermostated to the starting temperature of 30 °C and placed inside the coil. An optical fiber thermometer (Optocon AG, Weidmann Technologies Deutschland GMBH, Dresden, Germany) was used to measure the temperature of the sample at 3 s time intervals after switching on the field. Quantification of the heating power released by the particles was performed by the determination of the specific absorption rate (*SAR*, typically expressed in W/g of magnetic material), or power released by unit mass of magnetic material. This can be calculated as [41,42]:

(1) $$SAR=\frac{CV_{S}}{m}\frac{dT}{dt}$$

where *C* is the volume specific heat capacity of the sample (4185 J/LK for dilute aqueous suspensions), *Vs* = 0.5 mL is the sample volume, *m* is the mass of magnetic material (5 mg in all samples), and dT/dt is the initial slope of the temperature vs. time curve.

### 2.3. Cell Culture Assays

Human embryonic kidney (HEK 293; ECACC no. 85120602), human cervix adenocarcinoma (HeLa; ECACC no. 93021013ATCC), and human liver carcinoma (HepG2; ECACC no. 85011430) cells were supplied by the Cell Culture Facility (University of Granada). Cell lines were grown at 37 °C in a 5% CO_2_ atmosphere in Dulbecco’s Modified Eagle’s Medium (DMEM) supplemented with 10% (*v*/*v*) fetal bovine serum (FBS), 2 mM glutamine plus 100 U/mL penicillin and 0.1 mg/mL streptomycin.

### 2.4. Hyperthermia Effects on Tested Cells

Adherent growing cells were trypsinized following standard procedures and resuspended in Krebs Henseleit Buffer containing 20 mM glucose at 5 × 10^5^ cells/mL. FR positive (HeLa, HepG2) and control (HEK 293) cells (2 × 10^5^ cells) were incubated under constant shaking with 75 or 150 µg/mL of the different compounds for 2 h at 37 °C. Then, cells were subjected to MFHT (12 kA/m, 175 kHz) for 2 h, while maintaining a temperature of 37 °C for cell growth. Cells kept in exactly the same conditions without the application of the magnetic field were used as controls. To address the effects of hyperthermia, cytotoxicity and clonogenic assays were performed. 

### 2.5. Cytotoxicity of Compounds 

For cytotoxicity assays, 1 × 10^3^ cells were seeded in 48-well plates in quadruplicate immediately after magnetic field treatment and the cytotoxicity was assayed 48 h later by determining the percentage of cell viability (with respect to unexposed cells) using the 3-(4,5-dimethylthiazol-2-yl)-2,5-diphenyl-2H-tetrazolium bromide (MTT) method, which correlates the cellular metabolic activity with the number of viable cells in the culture. Results are reported as % viability based on the untreated control cells at 48 h, normalized to 100% viable. 

### 2.6. Clonogenic Assay

To study the differences in the ability to generate clones between untreated and treated cells, 1 × 10^3^ cells were seeded in 6-well plates immediately after the magnetic field treatment. After 72 h, cells were fixed with 2% (*w*/*v*) paraformaldehyde in PBS solution and stained with 0.01% (*w*/*v*) of crystal violet for 30 min. The number of clones was measured using ImageJ software (NIH). 

### 2.7. Statistical Analysis of Cell Tests

Results were expressed as means ± 1 standard deviation. The statistical significance of variations was evaluated using a two-way ANOVA. Post ANOVA pair comparison between the means of each group was performed by means of the Tukey HSD test in order to check for significantly different effects [43]. A *p*-value < 0.05 was considered significant. 

## 3. Results and Discussion

### 3.1. Particle Characterization

We hypothesize that the one-pot solvothermal synthesis of MNPs from FeCl_3_·6H_2_O, ethane-1,2-diol, sodium acetate, and the polyamine polymer PEI is a feasible approach to obtain amino-decorated MNPs suitable for hyperthermia and for further functionalization. In fact, the role of ethane-1,2-diol as a reducing agent and of sodium acetate as both an electrostatic stabilizer (to prevent particle agglomeration) and a coadjuvant in the reduction of FeCl_3_ is well established [37]. The use of PEI during the synthesis [34,35,44] or post-synthesis [32,33,37] to yield MNPs bearing amino groups has already been reported. 

Three different molecular weights of branched PEI were assayed to yield **MPN-1** (PEI 1.8 kDa), **MNP-2** (PEI 10 KDa), and **MNP-3** (PEI 25 kDa) that, after exhaustive washing with water, remained in suspension without sedimentation. The hydrodynamic diameters of **MNP-1**, **MNP-2**, **MNP-3**, and **MNP-4** were evaluated by DLS as 190 ± 50 nm, 160 ± 40 nm, 250 ± 140 nm, and 220 ± 60 nm, respectively, larger than the 85 nm reported by Wang et al. [35] for similar particles obtained with PEI10K. In all cases, the coating of the magnetite particles with PEI yielded highly stable systems, with visually no particle settling even several days after preparation. As an indication of the role of electrostatic repulsion between the particles on the observed stability, we determined their electrophoretic mobility in water. No attempt was made to calculate the zeta potential, as the mere notion of this quantity is not fully justified for particles coated with a polymer layer. The values of the electrophoretic mobilities (determined always by triplicate, using 9 determinations in each sample) were (in practical units of μm·s^−1^/V·cm^−1^): 2.528 ± 0.004, 2.738 ± 0.010, 3.339 ± 0.004, and 2.583 ± 0.005, for samples **MNP-1**, **MNP-2**, **MNP-3**, and **MNP-4**, respectively. These values indicate that the particles experience sufficient repulsive forces to account for their stability in addition to the steric repulsion associated to the polymer layer.

Samples **MNP-1** and **MNP-3** were further characterized by the high-resolution transmission electron microscopy (HRTEM). Micrographs in Supplementary Information File Figure S1 shows that the **MNP-1** particles are more regular and better defined than those of **MNP-3,** and that, although both samples show a selected area electron diffraction with an interplanar spacing of 0.25 nm (consistent with that of (311) planes in magnetite [45]), the crystallinity of **MNP-1** is superior. 

**Figure 1:**
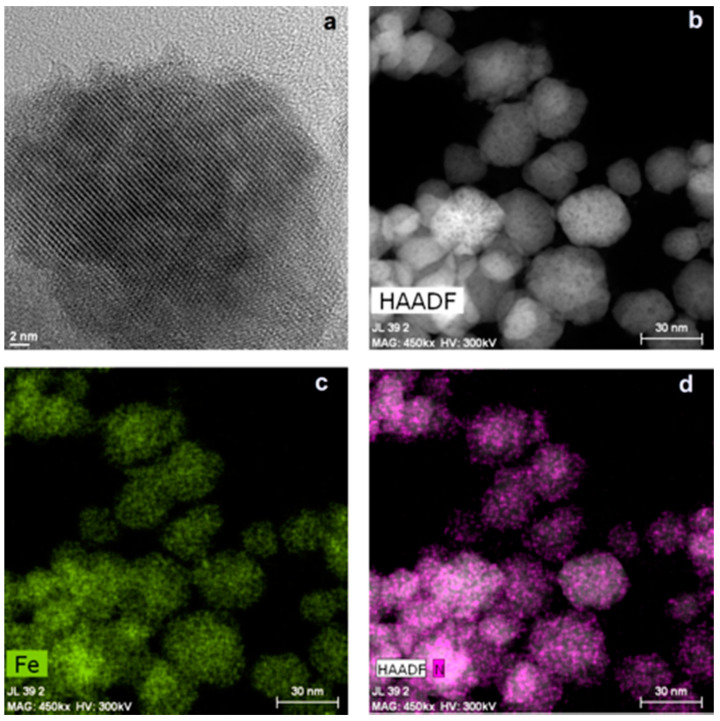
HRTEM (**a**) and high-angle annular dark-field (HAADF) images of **MNP-1** (**b**), showing the distribution of Fe (**c**) and N (**d**) atoms.

The HRTEM also allowed the measurement of the lattice spacings of 0.45 nm and 0.30 nm for **MNP-1** and 0.29 nm for **MNP-3,** compatible with the planes (111) and (220) of magnetite (Figure S2). Since sample **MNP-1** shows better magnetic parameters (see below) and crystallinity, efforts were focused on its characterization. The size of the electron-dense nuclei was estimated as 25 ± 6 nm, with 0.22 as the coefficient of variance (Figure S2), and the HRTEM revealed the crystalline order of the particles (Figure 1a).

In order to gain additional insight into the distribution of Fe and N in the nanoparticle, a high-angle annular dark-field imaging analysis was carried out, revealing that both Fe and N are uniformly distributed over the nanoparticle, with Fe forming the core, and N appearing grouped into spots on the surface (Figure 1b–d and Figure S3). These results suggest that the incorporation of PEI to the MNPs does not disrupt the crystal lattice of the magnetite, and that the amino groups are arranged as a set of patches on the surface.

**Figure 2:**
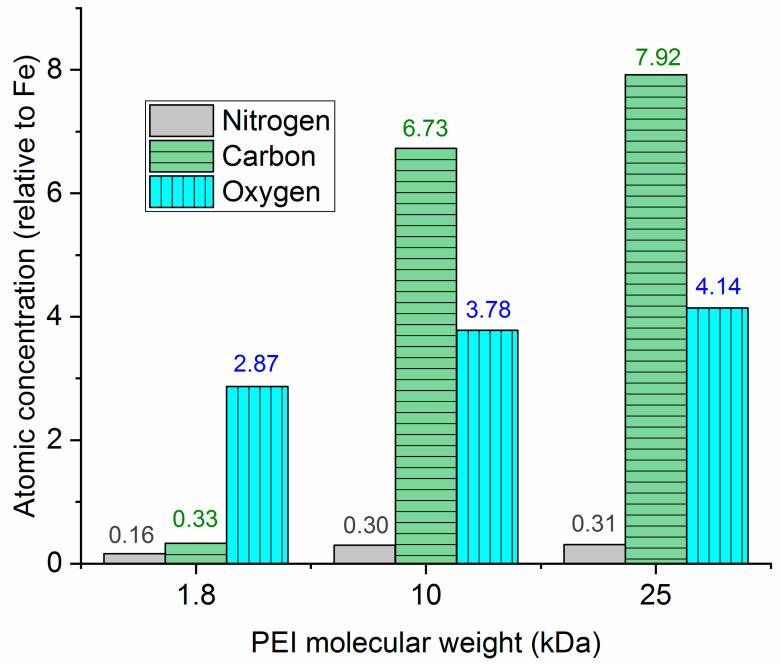
Relationship between the molecular weight of PEI and the relative atomic concentration of N (grey, no pattern), C (green, horizontal pattern), and O (cyan, vertical pattern). Values are normalized by the atomic concentration of Fe. The values of the normalized concentrations are indicated for clarity in performing a quantitative comparison.

The full scan XPS spectra (Figure S4, Table S1) present peaks at 56 eV, 285 eV, 399 eV, 530 eV, and 711 eV which are attributed to Fe3p, C1s, N1s, O1s, and Fe2p, respectively, confirming the incorporation of PEI and allowing the quantification of the elements present in the MNPs. The influence of the size of PEI on the composition of MNPs becomes apparent when the elemental analysis by the XPS is normalized by the abundance of Fe. Thus, the [O]:[Fe] and [C]:[Fe] ratios increase as the molecular weight of PEI becomes larger, whereas the [N]:[Fe] ratio for PEI10K and PEI25K remains very similar and twofold higher than that for PEI2K (Figure 2). The high resolution Fe2p spectra (Figure S5) present two major peaks at 710 eV and 723 eV, corresponding to Fe2p_3/2_ and Fe2p_1/2,_ respectively [46]. The Fe2p_3/2_ signal was fitted with two peaks at 709.7 eV and 711.0 eV that are consistent with the literature values for magnetite Fe^2+^2p_3/2_ and Fe^3+^2p_3/2_ [40]. The O1s spectra (Figure S5) were fitted with three peaks centered at 529.4 eV, 531.2 eV, and 533.3 eV, assigned to Fe–O, hydroxide, or defective oxide and organic oxide, respectively [47]. The high resolution C1s spectra (Figure S6) can be fitted with three peaks at 284.7 eV, 285.5 eV, and 288.5 eV that are assigned to C–C, C–N, and C = O, respectively, and the N1s signal centered at 399 eV was fitted with two peaks separated by 1.2 eV, pointing to the existence of N in two different environments [48]. 

### 3.2. Magnetic Properties and Magnetic Field Hyperthermia (MFHT)

**Figure 3:**
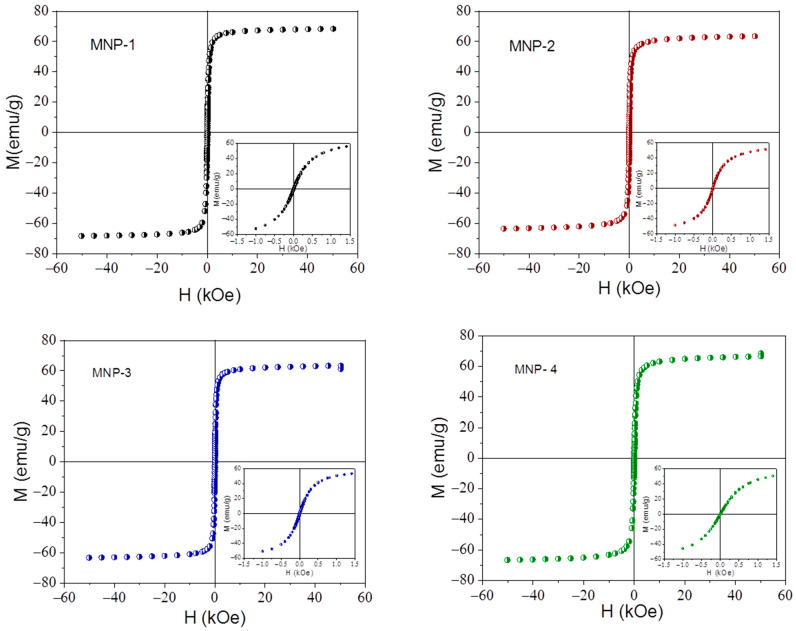
Magnetization cycles of samples **MNP-1**, **MNP-2**, **MNP-3**, and **MNP-4**. Insets: Low-field regions.

In order to evaluate their application in MFHT, a magnetic hysteresis investigation was first performed. The results are shown in Figure 3, and demonstrate that all samples have superparamagnetic behavior, with no hysteresis. The saturation mass magnetization was estimated as 68.9 emu/g for **MNP-1**, 63.3 emu/g for **MNP-2**, and 62.7 emu/g for **MNP-3**. On the whole, these values are comparable to the highest values reported for magnetic nanospheres in, for instance, [49]. Since, as evidenced by Figure 3, the saturation of magnetization takes place for field strengths *H* ≅ 798 kA/m, and the experimental data on hyperthermia are acquired at much lower fields of 12 kA/m so that saturation is never reached, we may wonder what the significance of the reported MS data for magnetic hyperthermia is. In fact, the major hysteresis cycle would not be involved in power dissipation. Even the minor loop, also shown in Figure 3, can be neglected, as the coercivity observed is almost zero. As stated by Dennis and Ivkov [11], strict superparamagnetic behavior is undesirable for heat release. We also note that the hysteresis cycle is obtained at static conditions, while MFHT requires over 100 kHz ac fields. This leads us to consider the physics behind magnetic hyperthermia, and, first of all, the characteristic times for spontaneous magnetization inversion, driven by thermal fluctuations. Such a time scale must be short as compared with the measurement time, that is, about 10 μs; if this duration is shorter than the inverted one, the magnetic moment will apparently remain fixed between successive applied field inversions, leading to finite coercivity and remanence, hence to a finite area hysteresis cycle now leading to heating power release.

The most widely used approach for estimating the characteristic time of a given particle is the so-called *linear response theory*, originally proposed by Rosensweig [50]. It is assumed that the magnetization depends linearly on the field (this means low strength of the applied ac field), through a complex magnetic susceptibility, its imaginary component being associated to the finite time required for the magnetization to be reversed under the field oscillations. Two mechanisms are admitted for the magnetization relaxation: one is the so-called Brownian relaxation, in which the magnetization changes because the whole particle rotates inside the viscous liquid (viscosity $\eta_{m}$) where it is suspended; the second one, the Néel relaxation, corresponds to the inversion of magnetization inside the fixed particle. Its respective characteristic times are:

(2) $$\tau_{B}=\frac{3\eta_{m}V_{H}}{k_{B}T}$$

(3) $$\tau_{N}=\tau_{A}\left(\frac{\sqrt{\pi }}{2}\right)\sqrt{\frac{k_{B}T}{K_{eff}V_{M}}}\exp\left(\frac{K_{eff}V_{M}}{k_{B}T}\right)$$

where $V_{H}(V_{M})$ is the hydrodynamic (magnetic) volume of the particle, $\tau_{A}$ is a characteristic (attempt) time (taken in the order of 10^−9^ s), and $K_{eff}$ is the anisotropy energy density, related to the saturation magnetization and the anisotropy field (*H_k_*: the anisotropy field, which is the field strength that must be applied for reversing the magnetization, given by $H_{K}=\frac{2K_{eff}}{\mu_{0}M_{sat}}$, $M_{sat}$ being the volume saturation magnetization). In the case of magnetite, *K_eff_* ≅ 25 kJ/m^3^, *H_k_* ≅ 0.14 T, and for particles with 200 nm diameter, the relaxation times fulfill the condition $\tau_{B}=2.8\;\mathrm{ms}<<\tau_{N}$. According to the linear response model, an average relaxation time can be calculated as $\tau =\left(\tau_{B}\tau_{N}\right)/\left(\tau_{B}+\tau_{N}\right)\approx \tau_{B}$, and if the frequency *f* of the field is below the critical value $2\pi f\tau_{B}=1$ (or f<60 Hz), the randomization by the thermal inversion of the particle orientation (taking a shorter time than the measurement field period) will dominate the behavior and no net magnetization will be produced, thus no power dissipation. 

If only the Brownian relaxation could be invoked for heating by ac magnetic fields, the power release would roughly depend only on the size of the paramagnetic particle and its viscous friction with the medium. Since this is not the case, and there is a tremendous variability in SAR values [51], more factors must be considered, the most important of which is anisotropy (including magnetic, shape, or aggregation). In fact, the power released is found to increase with the saturation magnetization and with $K_{eff}$. This gives us clues on the ways to increase the hyperthermia response [11].

**Figure 4:**
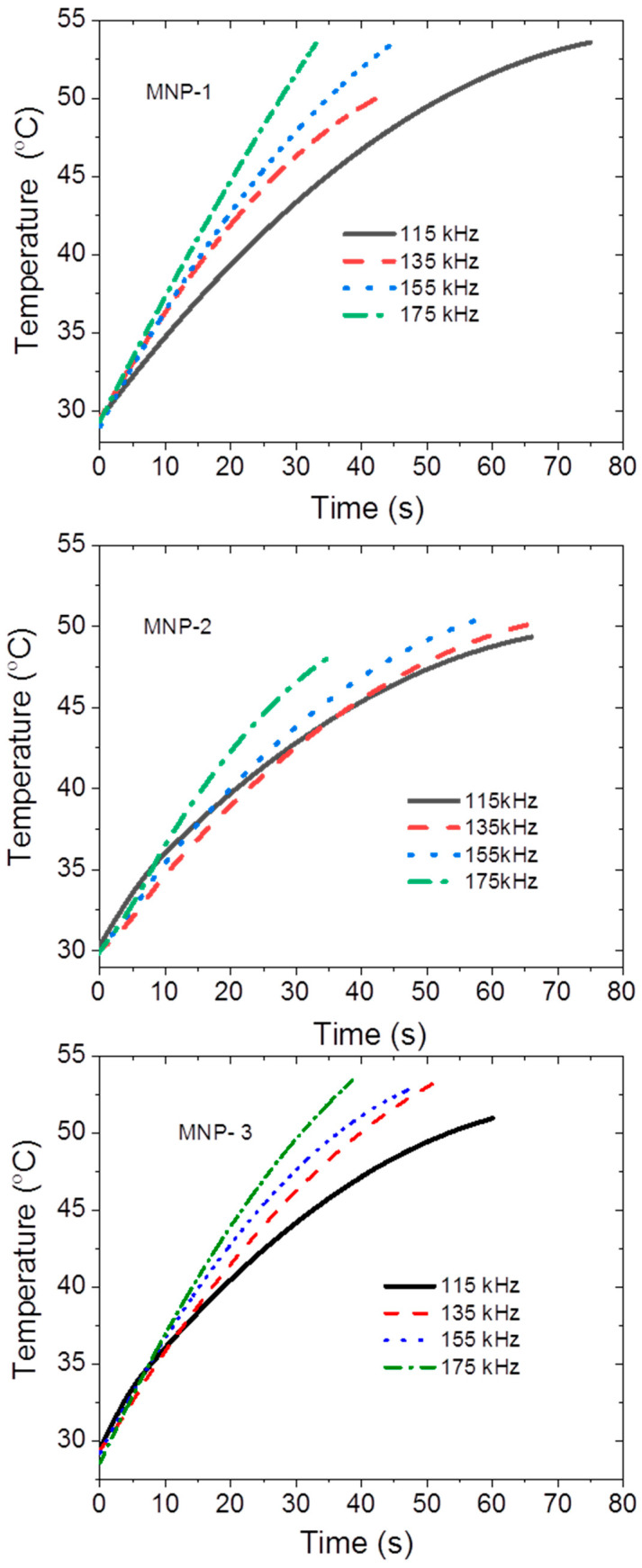
Time dependence of the temperature of the 10 mg/mL suspensions of the investigated nanoparticles after switching on a magnetic field with an amplitude of 12 kA/m and the frequency indicated.

Figure 4 shows the temperature variation of suspensions containing 10 mg/mL of magnetic nanoparticles with time of application of a 12 kA/m AMF of frequencies between 115 kHz and 175 kHz, which are below the safety limits of *H* × *f* < 485 kA/m.kHz [52], and within the range of those used by the few existing commercial devices. After 30 s at 175 kHz the temperature reached 51.6 °C, 41.1 °C, and 50.0 °C for **MNP-1**, **MNP-2**, and **MNP-3**, respectively (Figure 4), indicating a good performance in hyperthermia. This suggests that **MNP-1** is the best candidate for MFHT applications. Interestingly, **MNP-1** is the sample showing the largest magnetic saturation (Figure 3), in accordance with theoretical predictions. Quantification of the hyperthermia performance can be done through SAR calculations, as displayed in Table 1. Although all samples perform similarly, **MNP-1** yields slightly better values. On the whole, the SAR obtained ranges above the average of the values reported in a systematic investigation using 30 types of IONPs by Lanier et al. [51], and it is comparable to that reported by Mohapatra et al. [49] f or spheres. 

### 3.3. Functionalization of MNP-1

**Figure 5:**
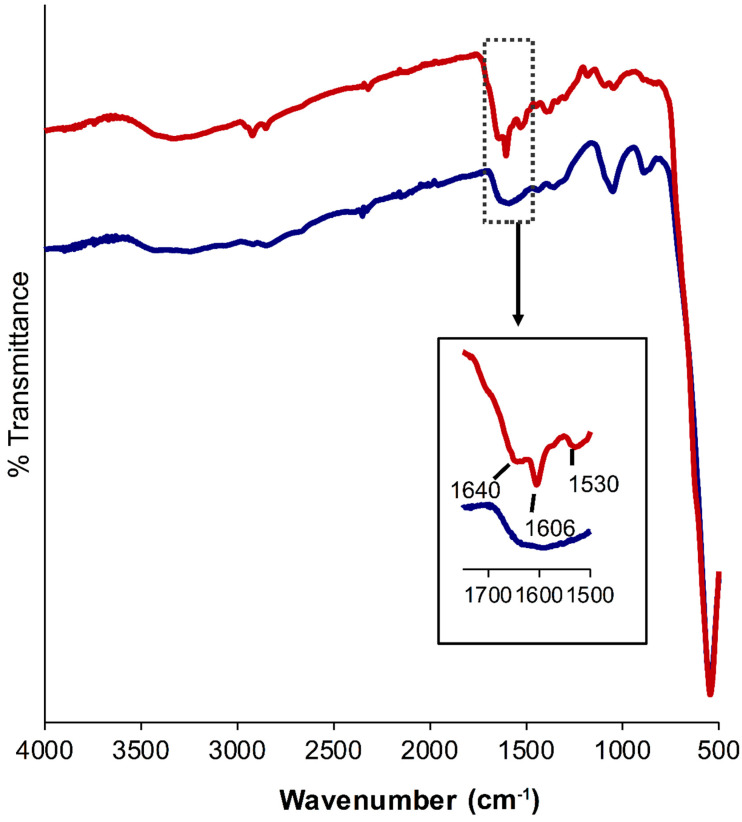
FTIR of **MNP-1** (blue) and after reaction with folic anhydride to yield **MNP-4** (red). Inset: detail of the significant peaks in the C = O stretching region.

One of the major challenges for the clinical use of MFHT is to achieve a significant and homogeneous temperature increase at the tumor while the surrounding tissue remains unaltered. The existence of a strong correlation between tumor size, temperature enhancement, and SAR has guided the synthesis of MNPs with improved SAR values [52]. However, the increase of temperature is also dependent on the concentration of the MNPs in the tumor tissue, and in consequence, the MNPs have been functionalized with different ligands to promote specific interactions with the target cells, with the aim of delivering and concentrating the MNPs at the site of action. As mentioned, FR is overexpressed in many cancers and this has been exploited for active cancer targeting, hence FA was selected as the ligand to functionalize **MNP-1**. In fact, examples of functionalization of MNPs with FA either by ionic interactions [26] or by covalent bonds by complex procedures, including click chemistry [22] and reductive amination [20] or coupling chemistry [21,23,24,25,27,28], can be found in the literature. Recall that the surface of **MNP-1** shows patches with N (Figure 1d), which according to the 1 Ns XPS spectra, correspond to amino groups (Figure S6). Since FA presents two carboxylic groups that can be transformed into an anhydride group [28], we envisioned the reaction of the folic acid anhydride with **MNP-1** to form an amide linkage as a straightforward strategy for the covalent functionalization of **MNP-1** with folic acid to yield **MNP-4**. FTIR spectrum of **MNP-4** presents signals at 1640 cm^−1^, 1606 cm^−1^, and 1530 cm^−1^ that correspond to amide I band, C = O stretching of the carboxylate, and amide II band, respectively (Figure 5). These features are in agreement with the reaction of **MNP-1** with folic anhydride to produce amide groups with the concomitant formation of carboxylate groups. The saturation mass magnetization was estimated as 60.6 emu/g for **MNP-4**, which is slightly lower than the 68.9 emu/g estimated for **MNP-1** (Figure 3), as expected from the coating with a significant layer of nonmagnetic material.

### 3.4. In Vitro Cell Viability Essays

Although the MNPs have been reported to be well tolerated by cells [53], high levels of iron are known to promote reactive oxygen species (ROS) via Fenton reaction, leading to ferroptosis, including lipid peroxidation, DNA damage, and cell death [54,55]. Indeed, concentrations as high as 31.36 mg of Fe/cm^3^ in tumors have been administrated intratumorally in patients with glioblastoma [56], but this mode of administration is not practical for metastasis, or large and/or poorly defined tumors. Intravenous administration is the most versatile method, the main challenge being the accumulation of the MNPs within the tumor. Feraheme^®^ (AMG pharmaceutical, Waltham, MA, USA) is among the FDA approved iron nanoparticle drugs that have been safely administrated in larger quantities, the recommended dose being 510 mg of iron [57]. In this context, the toxicity of **MNP-1** and **MNP-4** was evaluated against three cell lines that express different levels of FR, including the low-FR expressing HEK cells and the high-expressing FR HeLa and HepG2 cells [58,59]. The maximum concentration assayed was 150 µg MNPs/mL culture, which is within the range of the concentration of Fe in blood after administration of the recommended dose of Feraheme^®^, considering a 5 L blood volume. 

**Figure 6:**
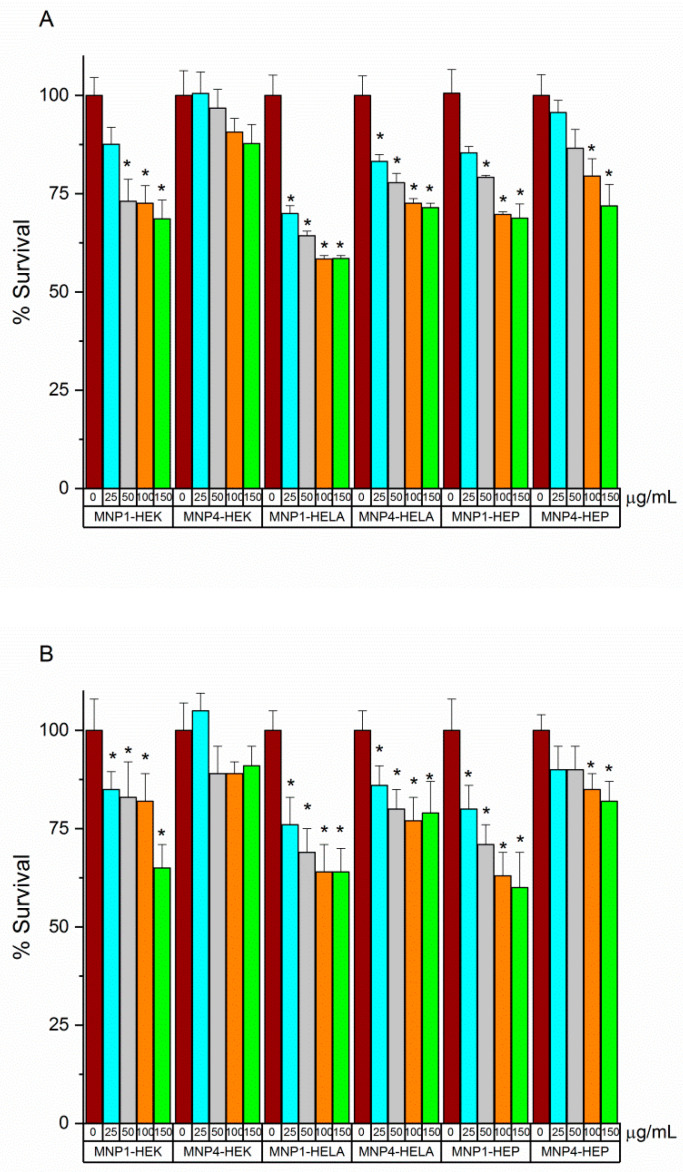
Cytotoxicity assays of **MNP-1** and **MNP-4** against HEK, HeLa, and HepG2 cells, incubated with increasing concentrations of the MNPs for 24 h. Results are means ± 1 standard deviation (*n* = 6). * *p* < 0.05 vs. control cells (incubated in the absence of nanoparticles). (**A**): 24 h incubation time; (**B**): 48 h.

Figure 6 shows that, in general, **MNP-1 and MNP-4** present a moderate cytotoxicity, the former being more toxic than the latter, in full agreement with the reported higher toxicity of positively charged nanoparticles [60]. The results obtained after 24 h (A) and 48 h (B) are very similar. This is in accordance with most studies in the literature, where the cytotoxicity tests are typically extended to 48 h at most [26,61,62,63,64].

**Figure 7:**
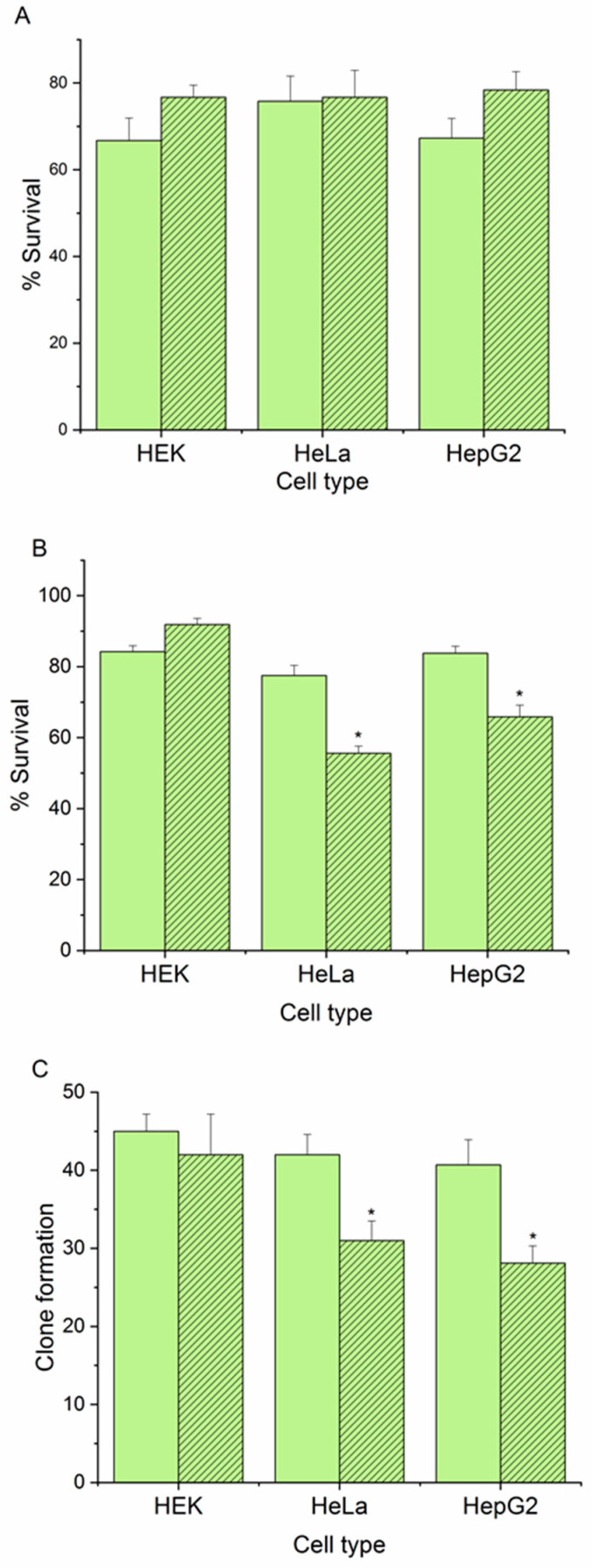
Cytotoxicity assays of **MNP-1** (**A**) and **MNP-4** (**B**) and clone formation of **MNP-4** (**C**) at 150 μg/mL against the low-FR expressing HEK cells and the high-expressing FR HeLa and HepG2 cells in the absence (full color) or presence (patterned color) of a magnetic field. Results are means ±1 standard deviation (*n* = 5). * *p* < 0.05 versus cells that were not subjected to the magnetic field.

An evaluation of the interest of **MNP-4** in MFHT was carried out at a concentration of 150 µg/mL on HEK (low FR overexpression), HeLa, and HepG2 cells (high FR overexpression) in a magnetic field of 12 kA/m and a frequency of 175 kHz, which, as mentioned, are reasonable parameters that are comparable to those used in the medical AMF devices (100 kHz and maximum of 15 kA/m, according to Magforce AG specifications (https://www.magforce.com/home/ Accessed on: 13 May 2021), and to those reported in most investigations [45]. Results are summarized in Figure 7, and as expected from the cytotoxicity evaluation, in the absence of a magnetic field, neither **MNP-1** nor **MNP-4** produce any significant cell toxicity or decrease in the capability to form colonies. The scenario changed when a magnetic field was applied, the effect on cell viability and colony formation being dependent on the expression of FR. Thus, whereas the effect of **MNP-1** is independent on the field, the action of **MNP-4** is dependent on both the expression of FR and the magnetic field. These results support the potential of **MNP-4** in MFHT at a concentration in the range of that approved by FDA for iron nanoparticle drugs since the cell viability and clone formation suffer a significant reduction in those cells expressing high levels of FR whereas the FR negative cells remain unaffected.

## 4. Conclusions

We have demonstrated that the one-pot solvothermal synthesis of magnetic nanoparticles (MNPs) from FeCl_3_·6H_2_O, sodium acetate, and ethane-1,2-diol in presence of polyamine polymer PEI is a reliable method to produce MNPs with amino groups on the surface, preserving the magnetite crystal lattice and allowing the coupling of different functions without any previous derivatization. It is interesting to mention that the method proposed provides, in a single stage, magnetite nanoparticles coated with PEI in a stable manner, which is probably advantageous over methods based on the post-synthesis addition of PEI on already formed particles, often requiring repeated layer-by-layer stages. 

Particles synthesized with PEI of three different molecular weights are all found to be superparamagnetic, with a magnetization saturation above 60 emu/g, which is comparable or higher than the values reported for spherical nanoparticles and make them attractive for MFHT. Studies performed for field frequencies 115, 135, 155, and 175 kHz and field strength *H* = 12 kA/m found that the heating power released ranges between 180 and 330 W/g, which is on the high side of reported values for these kinds of particles, and that PEI 1.8 kDa is the most suitable polyamine (sample **MNP-1**). Although larger rates of heat release have been reported, it must be mentioned that the real application of these particles in hyperthermia is not just based on rapid heating, but also on the reliability of their production, size, and the magnetic properties, control, and biocompatibility of the final magnetic nanostructure.

In addition, the particles are designed as elements of a so-called targeted thermal therapy. The combination of **MNP-1** with folic anhydride yields folic acid-functionalized nanoparticles **MNP-4**. In vitro cellular assays at a concentration in the range of that approved by FDA for iron nanoparticle drugs and conditions and a magnetic field comparable to those used in the medical AMF devices reveals that those cell lines with high levels of overexpression of FR are selectively damaged by **MNP-4** in the presence of the magnetic field, whereas those with a low level of expression of FR remain unaltered, supporting further evaluation of **MNP-4** on animal models. This is the expected continuation of this work, as the in vivo use of these particles will be the determinant for upgrading the synthesized nanostructures to therapeutic tools against cancer. They might furthermore become multiobjective tools if they can be also used as antitumor drug vehicles, whereby the thermal and chemical therapies can be applied at the same time in a target-selected fashion.

## Figures and Tables

**Table 1:** SAR values of the MFHT of the samples investigated. The uncertainties correspond to 95% confidence intervals in the slopes and the SAR.

Sample	Frequency	*dT*/*dt* (°C/s)	SAR (W/g)
**MNP-1**	115	0.48 ± 0.03	200 ± 12
	135	0.56 ± 0.04	233 ± 17
	155	0.67 ± 0.03	279 ± 13
	175	0.80 ±0.04	330 ± 17
**MNP-2**	115	0.38 ± 0.03	157 ± 12
	135	0.448 ± 0.026	186 ± 11
	155	0.48 ± 0.04	200 ± 17
	175	0.58 ± 0.04	239 ± 16
**MNP-3**	115	0.45 ± 0.03	187 ± 12
	135	0.58 ± 0.04	242 ± 17
	155	0.63 ± 0.04	261 ± 17
	175	0.72 ± 0.04	299 ± 17

## Data Availability

The data is available on reasonable request from the corresponding author.

## Supplementary Materials

The following are available online at https://www.mdpi.com/article/10.3390/polym13101599/s1, Figure S1: Dark field transmission electron microscopy image (a) and selected area electron diffraction (b) of MNP-1 (1) and MNP-3 (2). Figure S2: High resolution transmission electron microscopy image (a) and selected area electron diffraction (b) of MNP-1 (1) and MNP-3 (2). Figure S3: High-angle annular dark-field (HAADF) images of MNP-1 showing the distribution of Fe (a), N (b), Fe and N (c), and H (d) atoms. Figure S4: XPS spectra of samples (from bottom to top) MNP-1(blue), MNP-2(red), and MNP-3(black). Figure S5: High-resolution XPS spectra of Fe2p (a) and O1s (b) of MPN-1 (1), MNP-2 (2) and MNP-3 (3). Experimental data are shown in light blue, envelope in blue, and residuals (on top) in brown. Figure S6: High-resolution XPS spectra of C1s (a) and N1s1s (b) of MPN-1 (1), MNP-2 (2) and MNP-3 (3). Experimental data are shown in light blue, envelope in blue, and residuals (on top) in dark red. Table S1: Quantification (% atomic concentration) by XPS of the most significant peaks detected in **MNP-1**, **MNP-2** and **MNP-3**.
