# Supplementary material for: Poly(ethylene-imine)-Functionalized Magnetite Nanoparticles Derivatized with Folic Acid: Heating and Targeting Properties
Source: Polymers (Basel). 2021 May 15;13(10):1599. doi: 10.3390/polym13101599 (PMC8155902; doi:10.3390/polym13101599)
Supplement: Supplementary file 1 [file polymers-13-01599-s001.zip › polymers-1199311-SI.pdf]

Poly (Ethylene Imine)-Functionalized Magnetite Nanoparticles Derivatized with Folic Acid. Heating and Targeting Properties

Mariano Ortega-Muñoz [1], Simona Plesselova [2], Angel V. Delgado[3], Francisco Santoyo-Gonzalez [1], Rafael Salto-Gonzalez [2], Maria Dolores Giron-Gonzalez [2], Guillermo R. Iglesias[3,*] and Francisco Javier López-Jaramillo [1,*]

[1] Department of Organic Chemistry, Institute of Biotechnology, Faculty of Sciences, University of Granada, 18071 Granada, Spain; mortegam@ugr.es (M.O.-M.); fsantoyo@ugr.es (F.S.-G.)
[2] Department of Biochemistry and Molecular Biology II, Faculty of Pharmacy, University of Granada, 18071 Granada, Spain; s.plesselova@gmail.com (S.P.); rsalto@ugr.es (R.S.-G.); mgiron@ugr.es (M.D.G.-G.)
[3] Department of Applied Physics, Faculty of Sciences, University of Granada, 18071 Granada, Spain; adelgado@ugr.es
* Correspondence: fjljara@ugr.es (F.J.L.-J.); iglesias@ugr.es (G.R.I)

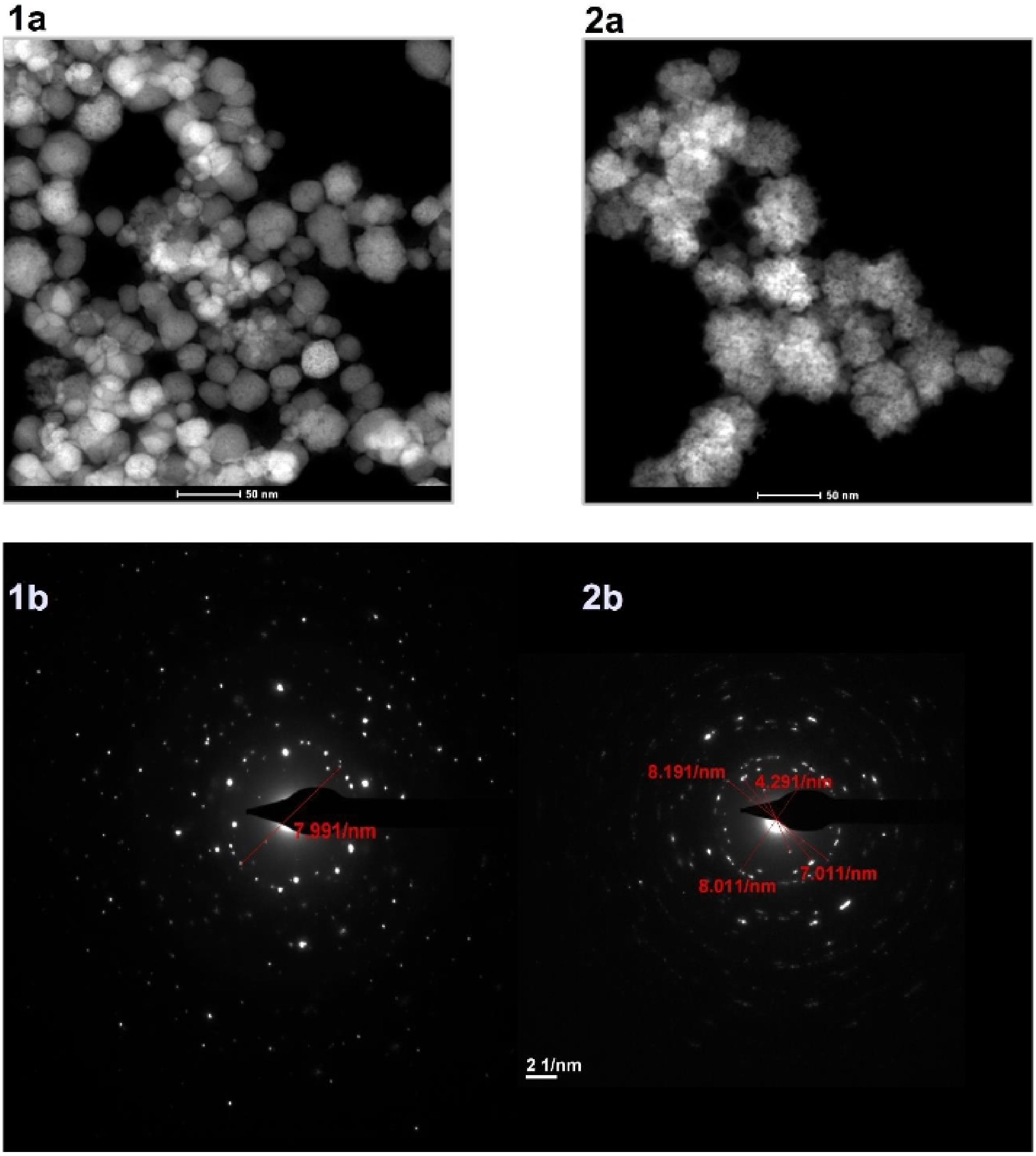

Figure S1. Dark field transmission electron microscopy image (**a**) and selected area electrondiffraction(**b**) of MNP-1(1) and MNP-3(2).

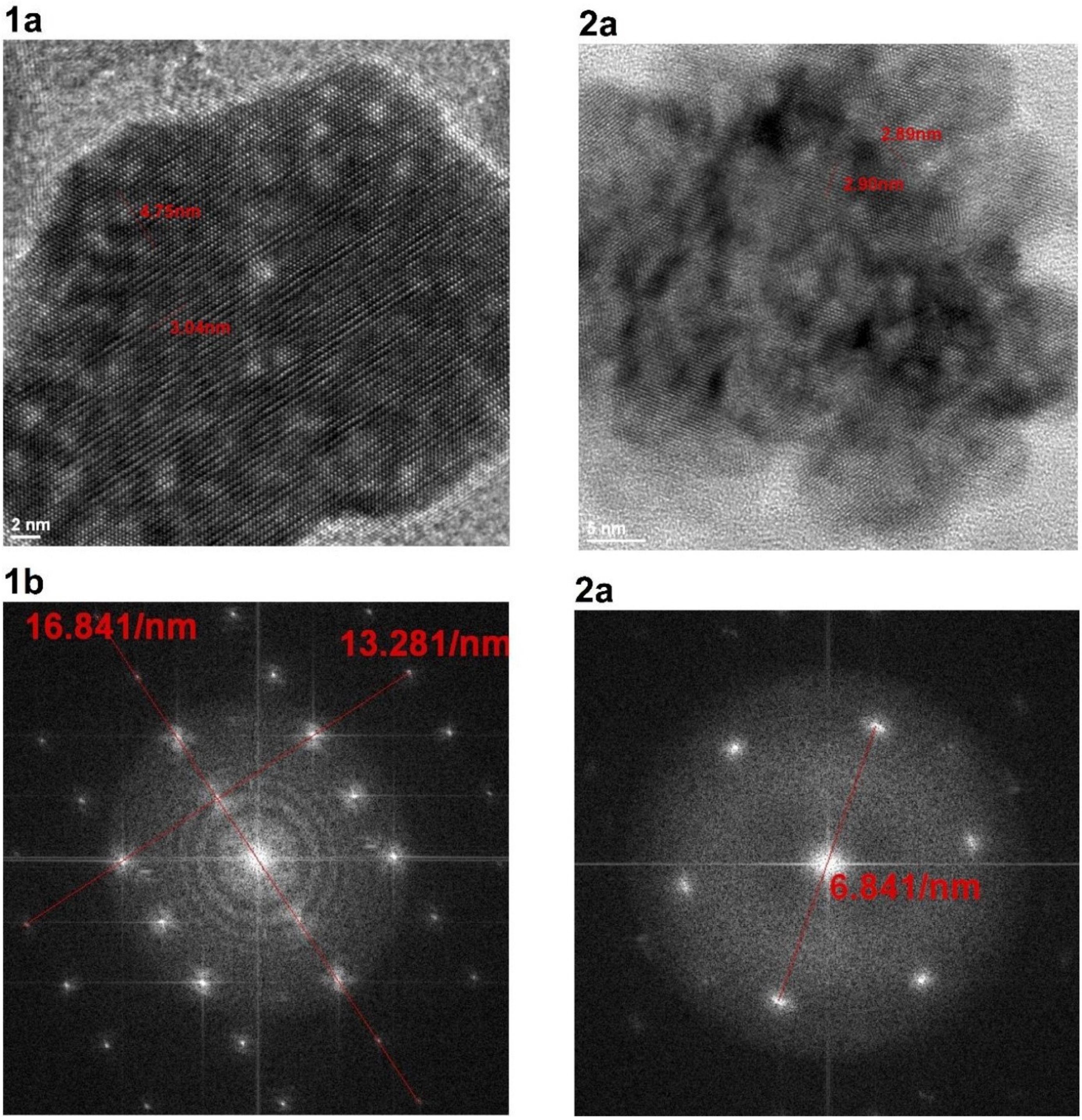

Figure S2. High resolution transmission electron microscopy image (**a**) and selected area electron diffraction (**b**) of MNP-1 (1) and MNP-3 (2).

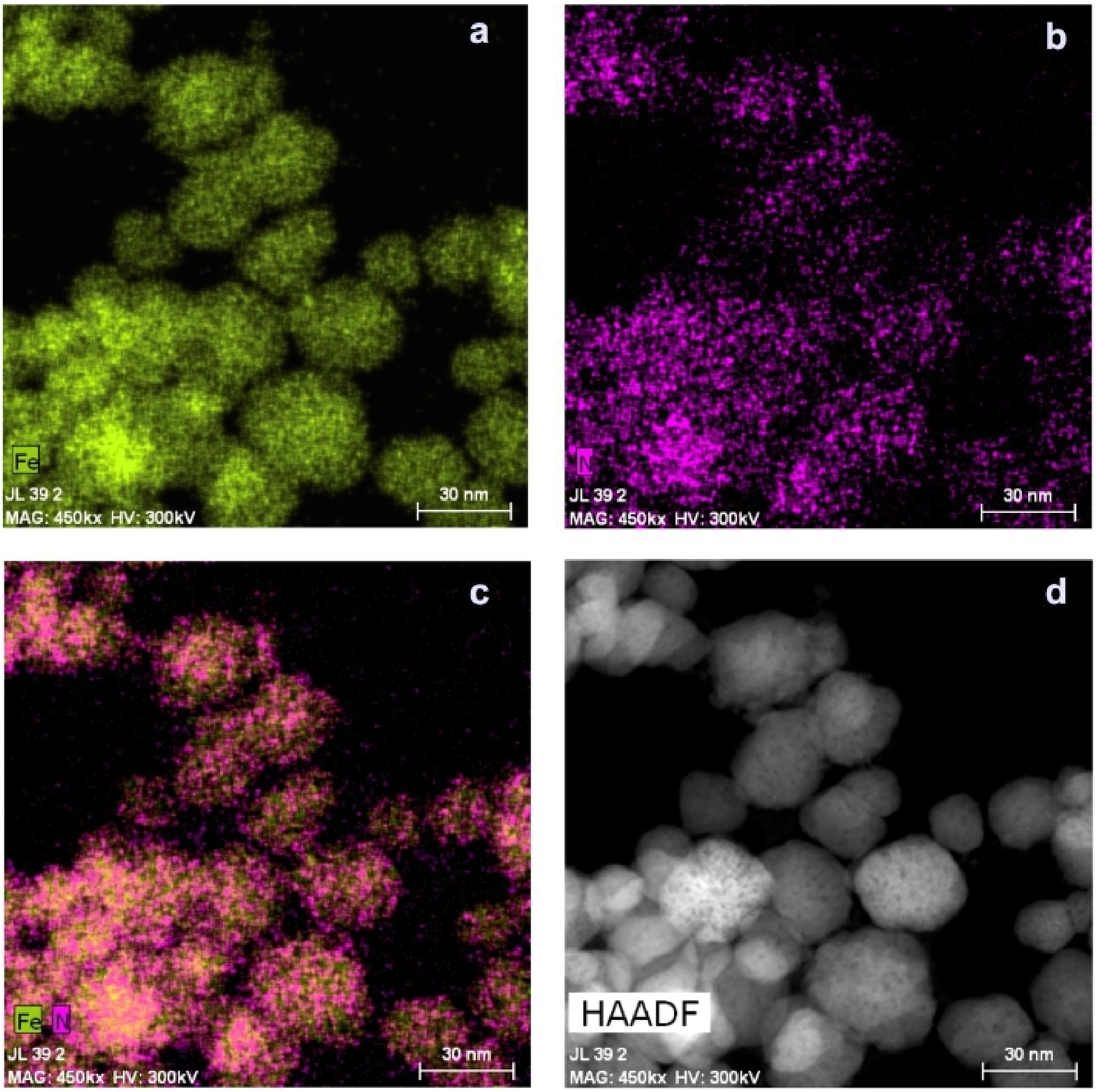

Figure S3. High-angle annular dark-field (HAADF) images of MNP-1showing the distribution of Fe (**a**), N (**b**), Fe and N (**c**), and H (**d**) atoms.

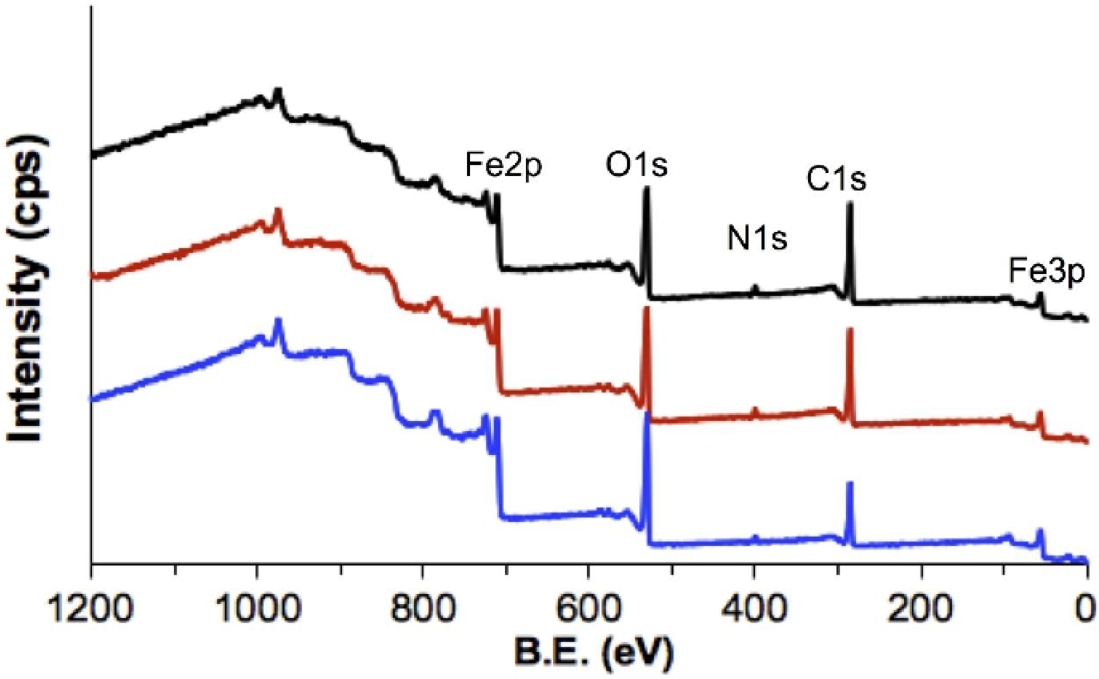

Figure S4. XPS spectra of samples (from bottom to top) MNP-1(blue), MNP-2(red), and MNP-3(black).

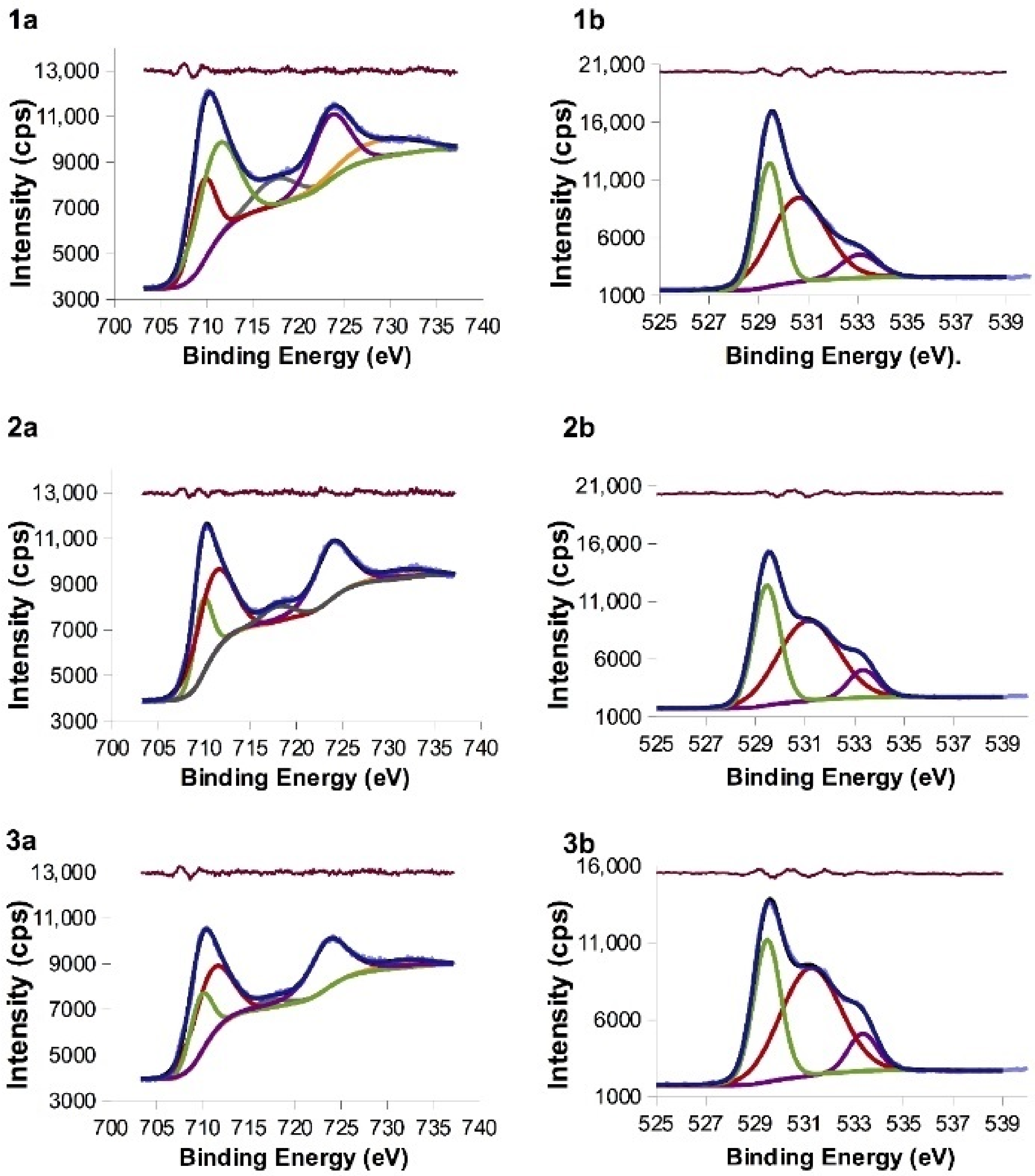

Figure S5. High-resolution XPS spectra of Fe2p (**a**) and O1s (**b**) of MPN-1 (1), MNP-2 (2) and MNP-3 (3). Experimental data are shown in light blue, envelope in blue, and residuals (on top) in brown.

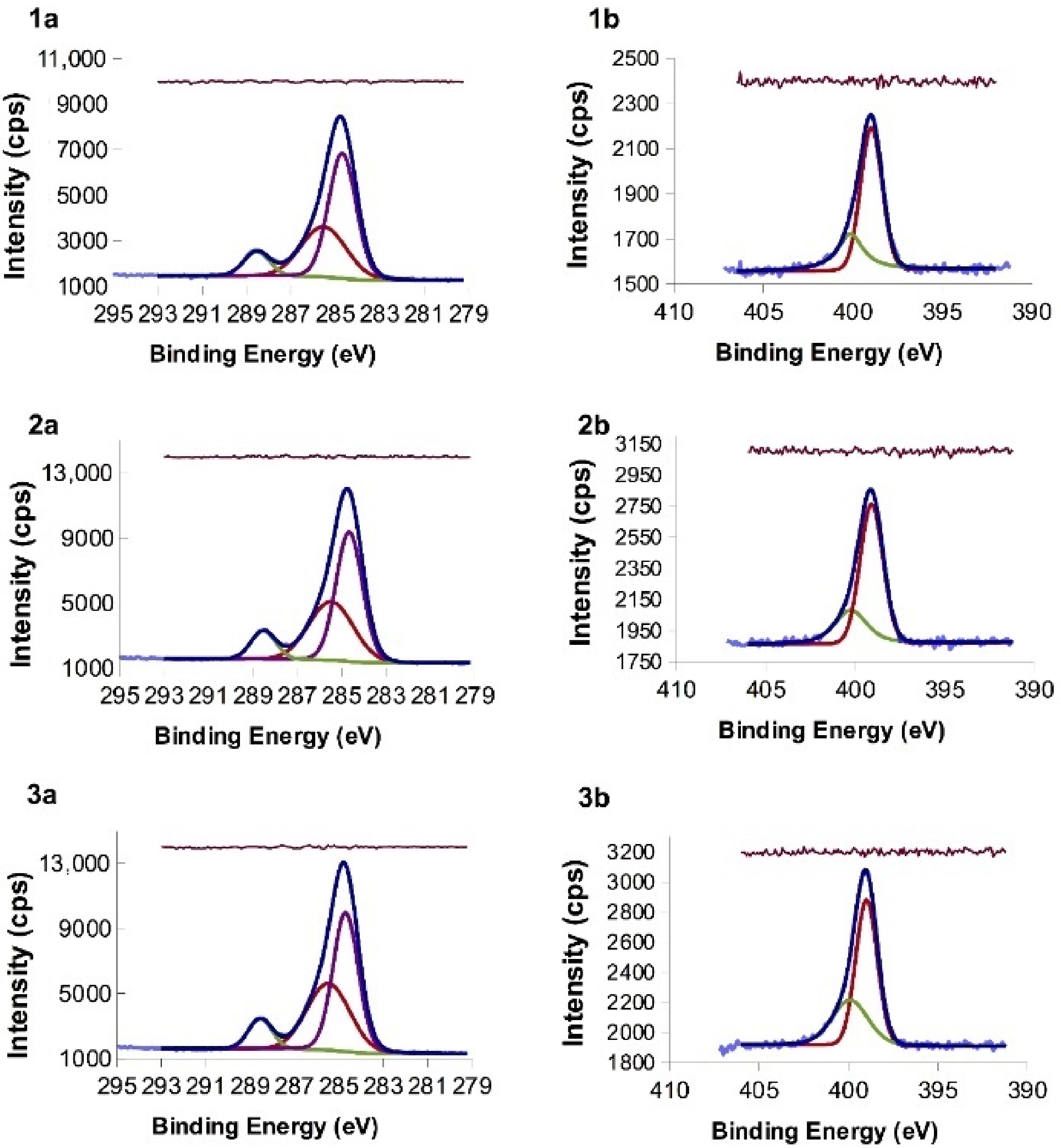

Figure S6. High-resolution XPS spectra of C1s (**a**) and N1s1s (**b**) of MPN-1 (1), MNP-2 (2) and MNP-3 (3). Experimental data are shown in light blue, envelope in blue, and residuals (on top) in dark red.

Table S1. Quantification (%atomic concentration) by XPS of the most significant peaks detected inMNP-1, MNP-2and MNP-3.

	**MNP-1**	**MNP-2**	**MNP-3**
%C1s	44.9	56.7	58.8
%O1s	38.6	31.8	30.8
%Fe2p	13.5	8.4	7.4
%N1s	2.1	2.5	2.3
